# A Preliminary Analysis on the Feasibility and Short-Term Efficacy of a Phase-III RCT on Mindfulness Added to Treatment as Usual for Patients with Chronic Migraine and Medication Overuse Headache

**DOI:** 10.3390/ijerph192114116

**Published:** 2022-10-29

**Authors:** Licia Grazzi, Alberto Raggi, Erika Guastafierro, Marco Passavanti, Alessia Marcassoli, Danilo Antonio Montisano, Domenico D’Amico

**Affiliations:** 1 Centro Cefalee, Fondazione IRRCS Istituto Neurologico Carlo Besta, 20133 Milano, Italy; 2 UOC Neurologia Salute Pubblica e Disabilità, Fondazione IRRCS Istituto Neurologico Carlo Besta, 20133 Milano, Italy

**Keywords:** mindfulness, chronic migraine, medication overuse headache, triptans, non-steroidal anti-inflammatory drugs, NSAIDs

## Abstract

This preliminary analysis of a single-blind phase-III RCT aims to compare the feasibility and short-term efficacy of mindfulness as an add-on to treatment as usual (TaU) in the management of patients with chronic migraine (CM) and medication overuse headache (MOH). Patients were randomized to either TaU (structured withdrawal of overused drugs, patient education and pharmacological prophylaxis) or TaU + MIND, wherein patients additionally received six 90 min weekly group sessions of mindfulness-based therapy. Repeated measures analyses were used to test whether patients in the two arms showed different course with regard to headache frequency and medication intake over a three-month period. Drop-out rates were not different between the two groups: 6/89 (6.7%) and 9/88 (10.2%) among those in TaU and TaU + MIND, respectively. A significant effect of time for all variables was shown, together with a significant effect of time by group, favoring TaU + MIND condition for headache frequency (*p* = 0.025) and NSAID intake (*p* = 0.007), controlling for age and CM duration. In total, 45/83 (54.2%) and 69/79 (75.9%) of the patients allocated to TaU and TaU + MIND, respectively, achieved 50% or more headache-day reduction (chi-squared 8.38, *p* = 0.004). Our preliminary analysis indicates that adding six mindfulness-based sessions to TaU was feasible and showed short-term efficacy in the treatment of patients with CM and MOH.

## 1. Introduction

Headache disorders constitute a heterogeneous group of neurological conditions whose common denominator is head pain. The International Classification of Headache Disorders, 3rd edition [1], distinguishes between primary and secondary headache disorders; the most common primary disorders are tension-type headache and migraine. According to the most recent estimates of the Global Burden of Disease Study, headache disorders are the second most prevalent condition among all-age subjects (and also among those aged 15–49), whereas in terms of disability, such conditions rank third among all-age subjects (and first among people aged 15–49), both globally [2] as well as in Italy [3]. While tension-type headaches are largely prevalent, and relatively disabling, migraines are associated with a higher level of disability [2,3].

Chronic migraine (CM) is the negative evolution of episodic migraine (EM), and it is characterized by 15 or more headache days per month for more than 3 months, of which at least 8 per month have typical migraine features [1]. It is often associated with the overuse of acute medications; in this case, CM presents a comorbidity with a secondary headache disorder, medication overuse headache (MOH) [1]. MOH is characterized by 15 or more headaches per month for more than 3 months in patients with pre-existing primary headaches (most often CM), and it develops as a consequence of regular medication overuse but usually resolves once the overuse is stopped. MOH prevalence ranges from 1 to 2% in the general population [4] and it is associated with poor quality of life (QoL), significant disability, and societal burden and cost [5,6].

Migraines arise out of the interaction between biological mechanisms, environmental and lifestyle issues, and psychological factors [7]. Examples of the first include genetic predisposition and comorbidities [8], which support the use of medications for prophylaxis and acute treatment. Examples of the second include, for example, inadequate sleep and eating habits, and limited level of physical activity [9,10,11], which support the need for lifestyle interventions. Finally, among psychological factors, examples include high levels of rigidity and obsessiveness, pain catastrophizing and relevant levels of anxiety and depression (also beyond clinical comorbidity) [8,12,13], which support the need for behavioral interventions.

Given the intrinsic biopsychosocial feature of migraine disorders, and of CM-MOH in particular, and the fact that these patients not only experience relevant pain, but also important limitations in QoL, daily activities, and disease burden [14], treatment has to include both pharmacological [15,16] and non-pharmacological options [17], as well as patients’ education. Among non-pharmacological ones, mindfulness has gained attention in recent years. Some reviews showed its efficacy for the management of migraines and other severe headaches [18], and suggested that its effect is comparable to that of pharmacological treatments [19].

### 1.1. An Overview of Studies Addressing Mindfulness in Headache Disorders

Several studies have been published in recent years that address the use of mindfulness in headache disorders. The majority of these studies were, however, on mixed populations of patients with EM and CM together [20,21,22,23], or EM only [24,25,26], whereas other studies mixed patients with different kinds of primary headaches [27,28,29,30,31,32,33]. To the best of our knowledge, few studies from our group have addressed mindfulness in patients with CM-MOH [34,35,36,37].

Almost all studies performed a comparison against no treatment, treatment as usual (TaU) or waitlist [20,21,22,23,24,25,26,27,28,29,30,31,32,33,34,35,36], with the exception of one study that was based on a single group [37]. Headache frequency was used as an outcome of treatment in almost all studies [20,21,22,24,25,26,34,35,36,37], with the variation over time being significant in the majority of the studies [21,22,25,26,34,35,36,37]. A superior reduction in migraines compared to the control condition was found by Simshäuser and colleagues [26], who compared the longitudinal course of headache frequency between patients allocated to receive mindfulness-based cognitive therapy adapted for migraines against waitlist patients. The remaining studies generally addressed measures of perceived pain, QoL, impact or disability as the main outcomes [23,27,28,29,31,32,33].

Finally, two studies addressed the use of acceptance and commitment therapy (ACT), which incorporates mindfulness practice as part of the treatment. The first showed a superior effect on headache frequency reduction in patients receiving TaU + ACT compared to those receiving TaU only [38]. The second study addressed disability and QoL as the main outcomes, and showed the relevance of headache acceptance, cognitive defusion, avoidance of headache, and mindfulness as mediators toward improvement in disability and QoL [39].

### 1.2. Study Aims

In sum, available studies that compared mindfulness alone to education or standard treatment have shown similar outcomes with respect to headache frequency. However, to date, it is difficult to address the efficacy of mindfulness due to inadequate sampling in available studies, and because the majority of them relied on a mixed population and did not employ the randomization of participants. Thus, randomized controlled trials (RCTs) are needed to address mindfulness efficacy in patients with CM and MOH. In addition to this, many studies compared mindfulness against waitlist, i.e., to non-treatment, which is clearly not what would be undertaken in standard practice. As previously stated, migraines (including CM-MOH) require a biopsychosocial approach in which biological mechanisms, environmental and lifestyle issues, and psychological factors are jointly addressed [7]. In a pilot non-randomized study, we showed that the 12-month course of headache frequency and medication intake in patients with CM and MOH, was similar between patients under pharmacological prophylaxis only and patients attending six mindfulness-based sessions only [34]. Therefore, we hypothesized that adding on mindfulness to treatment as usual (TaU), i.e., withdrawal of overused drugs, education, and pharmacological prophylaxis, may lead to a superior improvement, rather than prophylaxis alone.

The aim of this paper is to present a preliminary analysis on the feasibility and short-term (three months) efficacy of a mindfulness-based protocol as an add-on to TaU in a group of patients with CM and MOH who participated in a single-blind phase-III RCT.

## 2. Materials and Methods

### 2.1. Study Design and Sampling

The MIND-CM study was designed to test the superiority of a six-week mindfulness-based protocol (the TaU + MIND condition) as an add-on to TaU, i.e., considering withdrawal, prescription of a tailored prophylaxis, and patients’ education. The study was approved by the ethical committee of our Institute (approval no. 51/2018) and was registered on clinicaltrials.gov (NCT03671681). The trial was conducted according to the original protocol.

The sample size was designed with the hypothesis that 48% of patients allocated to TaU would achieve ≥50% headache-day reduction after 12 months, and that adding mindfulness might increase this figure by 20%. We set alpha to 0.05 and power to 80%, and determined that 75 patients per group were needed in each group (randomization 1:1); considering that up to 12% of patients might be lost at follow-up, we determined that 170 patients should be randomized. For this preliminary analysis report, we addressed the three-month follow-up.

A computer-generated list of random numbers was used to create a simple randomization list with no restrictions, so that each patient had a 50% probability of being included in either the TaU or TaU + MIND group. The study neurologist who enrolled the patients (D.D.) was not involved in the randomization of patients to the two groups and remained blind to the attendance of patients to mindfulness sessions. A single researcher (A.R.) prepared the randomization list and a set of opaque envelopes two months before the beginning of the study, and together with two other researchers (E.G. and M.P.), randomized patients to the two groups and handled data collection. None of these researchers were involved in the mindfulness session administration, which was on the contrary handled by another study neurologist (L.G.) who had undergone extensive training in mindfulness at the Association for Meditation and Awareness under the supervision of Prof. Corrado Pensa, and is a leading expert in mindfulness and other behavioral treatment for headache disorders.

After the screening visit, on the second day of withdrawal, patients meeting the selection criteria were asked to enter the study on a voluntary basis. Those who accepted signed an informed consent form and were randomized to the two groups. Randomization was performed at that point by opening the opaque envelope. At each follow-up, documents referred to primary and secondary endpoints were collected by one of the three researchers (A.R., E.G. or M.P.), who reminded patients not to disclose their allocation (TaU or TaU + MIND) to the study neurologist who performed clinical evaluation.

### 2.2. Participants

Participants were adults meeting the criteria of the International Classification of Headache Disorders, third version [1] for CM (i.e., code 1.3) and MOH (i.e., code 8.2), who attended structured withdrawal in ward or in day hospital between November 2018 and November 2021, with follow-up being between February 2019 and February 2022. Exclusion criteria were: psychiatric comorbidities of the psychotic area; pregnancy; secondary headaches; any previous experience with mindfulness. Each patient signed the informed consent form prior to randomization.

### 2.3. Intervention

All patients underwent a treatment program either in a ward or day hospital setting which included: abrupt withdrawal of the overused symptomatic drugs; intravenous “bridge therapy” to avoid rebound headaches and withdrawal symptoms; education focused on the correct use of acute medications and on lifestyle issues, which encouraged physical activity, adequate hydration, regular meals, and sleep hygiene [40]. Tailored prophylaxis was prescribed by the treating neurologist on the basis of the clinical features and previous experience with pharmacological prophylaxis of each patient, and was based on a pharmacological compound for all patients and, if appropriate, on nutraceuticals (e.g., magnesium, Q10 coenzyme, or B2 vitamin).

On day two of the treatment program, patients were randomized to TaU or TaU + MIND, consisting of six sessions of mindfulness-based intervention, as previously described [41]. In brief, patients were instructed to assume a relaxed sitting position that enabled regular breathing and to close their eyes, to focus their attention on their breathing, on the present, and on silence to enhance awareness of current mind and body sensations, as well as on their thoughts, accepting them in a non-judgmental way. In the last sessions, they were invited to preserve themselves from interfering thoughts, and to focus on the present and on the sensations they receive from their bodies. They were finally encouraged to supplement their training with regular 7–10 min of daily home self-practice.

### 2.4. Measures and Data Analysis

In this preliminary analysis, we focused on the primary endpoint, i.e., headache frequency, and on the intake of acute drugs. We used means and standard deviations (SD) for continuous variables, and frequencies and percentages for categorical ones. Basic sociodemographic and clinical descriptions, i.e., patients’ age, sex, CM duration and previous structured withdrawals, were derived from clinical documents. The normality of distribution was checked using Q–Q plots.

We tested baseline differences between the two conditions for patients’ age, sex, CM duration, headache frequency, and acute drug consumption (total drug, triptans, non-steroidal anti-inflammatory drugs—NSAIDs) using the independent sample t-test and chi-squared test.

To address feasibility, we used the chi-squared test to compare the two groups in terms of drop-out rates (herein used as a proxy for protocol feasibility).

Short-term efficacy was tested in two ways on the subsample of study completers. First, we used the chi-squared test, comparing the two groups for the achievement of 50% or more reduction in headache frequency compared to baseline. Second, we used repeated measures ANOVA by group to address differences over the study period in the two groups for three-month headache frequency, NSAID intake, triptan intake and total medication intake. In the repeated measures ANOVA, we also entered age and CM duration as covariates.

Analyses were two-tailed, with *p* < 0.05, and carried out with SPSS 28.0 (IBM Corp. Released 2021. IBM SPSS Statistics for Windows, Version 28.0. Armonk, NY, USA: IBM Corp).

## 3. Results

**Figure 1:**
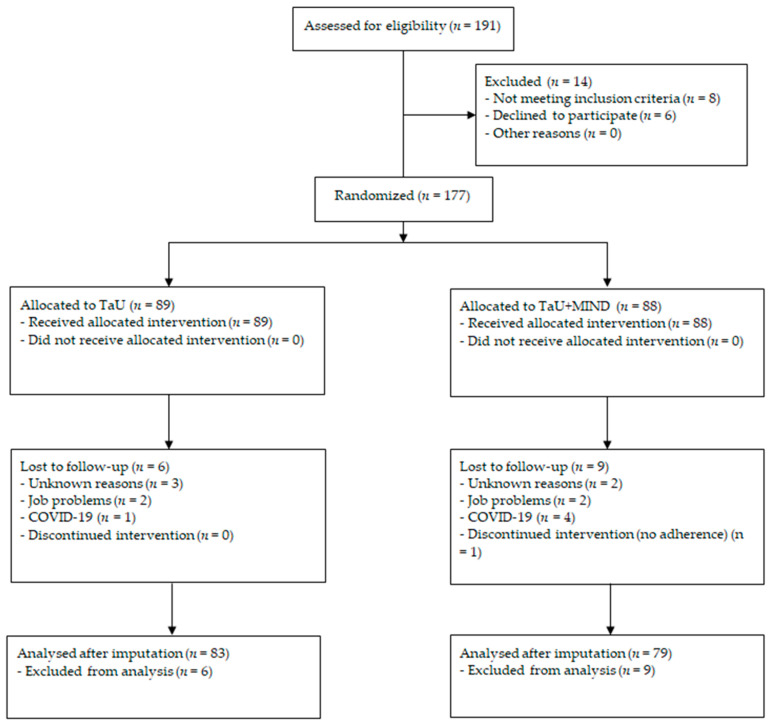
Study flowchart.

A total of 177 patients were randomized to the two conditions: 89 to TaU and 88 to TaU + MIND (see Table 1 for baseline features). Patients allocated to the TaU + MIND group showed a higher NSAID intake (*p* = 0.014; mean difference 31.0, 95% CI: 6.4–55.6 intakes/three months). Drop-out rates between the two groups were not different (chi-squared 0.69, *p* = 0.405): 6 out of 89 (6.7%) among those in TaU, and 9 out of 88 (10.2%) among those in TaU + MIND dropped out in the first three months. See Figure 1 for a study flowchart.

Repeated measures ANOVA (Table 2) showed an effect of time for all variables of interest (*p* < 0.001 for all repeated measures). In addition, a time by group effect favoring patients in the TaU + MIND group for headache frequency (*p* = 0.029) and NSAID intake (*p* = 0.005) was shown too. Once the analysis was repeated, keeping age and CM duration as covariates, the main results did not change significantly, with a time by group interaction favoring patients in the TaU + MIND group for headache frequency and NSAID intake (*p* = 0.025 and *p* = 0.007, respectively).

Finally, 45 out of 83 (i.e., 54.2%) of the patients allocated to TaU and 60 out of 79 (i.e., 75.9%) of those allocated to TaU + MIND achieved a reduction in headache frequency ≥50% compared to baseline (chi-squared 8.38, *p* = 0.004).

## 4. Discussion

The preliminary analysis of this RCT showed that, after three months from withdrawal, patients allocated to receive six weeks of mindfulness-based protocol as an add-on to TaU reported a higher decrease in headache frequency and NSAID intake than that observed among those in the TaU group, also controlling for age and CM duration. Specifically, patients allocated to the TaU + MIND condition experienced a 25.3% higher frequency of reduction in headache frequency ≥50% compared to baseline (which will be used as a primary endpoint for the 12-month analysis), with no differences in drop-out rates, thus supporting the feasibility of the protocol.

The decrease in headache frequency and NSAID intake, but not in total amount of acute drugs, was significantly higher among patients in the TaU + MIND group, also controlling for potential confounders such as age and CM duration. It has to be noted that these results were obtained even though the patients allocated to the TaU + MIND group showed higher NSAID intake, a discrepancy for which we do not have any specific explanation, other than a casual distribution. Conversely, the balance observed on the main outcome, i.e., headache frequency, enables us to hypothesize that the superior improvement in these two variables might depend on the specific mechanisms of the action of mindfulness.

In particular, mindfulness improves the ability to focus on the present, to accept daily difficulties, such as migraine headaches, to manage stressful situations, and to enhance self-efficacy [42]. With reference to headache pain, this likely meant learning to avoid the “pain-pill” automatism, which easily leads to the vicious cycle of pain and medication. The two groups achieved very similar results at three months from enrolment, i.e., an acceptable consumption of NSAIDs and of triptans. It has to be acknowledged that this evaluation was performed at three months from the structured in-hospital withdrawal, which acts on three aspects: withdrawal from overused drugs, prescription of tailored prophylaxis, and education on lifestyle and on the use of drugs. Achieving such results on drug intake should be the “normal” outcome in such a short period; in reports based upon three-month evaluation, we found that 72% to 84% of patients achieve successful withdrawal outcome by three months (i.e., acceptable headache frequency and/or absence of medication overuse [43,44]). Our results, in addition to reaching an acceptable level of NSAID and triptan consumption, also demonstrate that the reduction in NSAIDs was significantly larger. The fact that NSAIDs, and not triptans (i.e., migraine-specific drugs), were more widely reduced in these patients is indicative that the “pain-pill” automatism has been tackled. Patients allocated to TaU in fact reduced their average NSAID consumption per headache day by 0.2 pills per headache day (from 1.0 to 0.8), whereas those in TaU + MIND reduced intake by 0.6 pills per headache day (from 1.5 to 0.9), thus suggesting a higher ability to manage migraine headaches with no use of painkillers. The full study follow-up will show whether such a reduction will remain wider among patients allocated to TaU + MIND compared to those in the TaU group, i.e., whether the expectable relapse rates, which might involve 34% of the sample [45], will be lower among patients in the TaU + MIND group.

One of the most outstanding trials on mindfulness is the one carried out by Wells and colleagues [22], who showed that MBSR did not improve headache frequency more than headache education after 12 weeks. However, it has to be noted that our sample is quite different in terms of clinical severity (more than 20 headache days per month and MOH vs. 9.5–9.8 on average without MOH in the trial by Wells and colleagues), and mindfulness was used as an add-on to the control condition, whereas Wells and colleagues compared MBSR to education only. Similar results were reported in other studies [21,25,26,34,35,36,37], whereas a superior result arising out of time of treatment interaction was previously reported on samples of patients with episodic migraine by Simshäuser and colleagues [26], who administered a mindfulness-based cognitive therapy specifically adapted for migraine vs. waitlist, and by Grazzi and colleagues [38], who administered ACT as an add-on to TaU.

A note on drop-out rate has to be made, which is not negligible in such a short period. The reason for this lies in the outbreak of the SARS-CoV-2 pandemic in 2020 and the consequent lockdown, which prevented many patients from accessing our center. Telemedicine approaches were organized in our center to address the limited mobility imposed by the lockdown [46]; however, this took some time and unfortunately some patients were lost in the first months of the outbreak. Specific mindfulness-based approaches were also implemented and devoted to patients who could not be enrolled in the present trial [47].

The literature on adverse events (AEs) in mindfulness-based treatments is sparse and in its infancy [48,49,50]. A recent review by Binda and colleagues stresses AEs for meditation practice, including anxiety and pain, and more severe events such as psychosis or mania. However, these severe events seem to be associated with and confounded by the intensity and length of the meditative practices and psychological stressors, as well as the history of psychiatric disorders of those engaging in the practice [50]. This, however, neither corresponds to the profile of the sample herein enrolled, from which psychiatric comorbidities of psychotic area were excluded, nor to the kind of intervention that consisted of six sessions and 7–10 min of daily practice. Although we did not systematically collect AEs, what can be reported is that none of the patients abandoned the sessions due to severe psychological discomfort, and no relevant events, such as panic attacks, happened. Some patients, but this has to be taken as anecdotal information, reported feeling anxious during the sessions, and memories and thoughts related to past stressful experiences were occasionally reported too: the occurrence of this might be around in one of ten subjects. With regard to pharmacological prophylaxis, none of the patients experienced relevant symptoms that necessitated a cautious interruption of treatment or a modification at three months; however, again we did not implement a solicited reporting of AEs, and prescribed prophylactic treatment was selected among those authorized by the European and Italian Medical Agencies.

A note on the effect of COVID-19 on the trial has to be made. Approximately half of those who were lost to the 3-month follow-up and allocated to the TaU + MIND condition (4 out of 9) were lost due to COVID-19 infection. These patients were enrolled at the beginning of 2020, a time when the awareness of the disease was limited. The difference in the rates of COVID-19 as a reason for dropping out might be casual; however, we cannot exclude that a contagion pathway connected to the attendance of mindfulness sessions played a role.

Some limitations need to be considered. First, the study was based in a single center with patients who were among the most severe ones. Second, the two arms were unbalanced with regard to NSAID intake. Third, it has to be taken into account that the MIND-CM trial was designed to address the superiority of TaU + MIND over TaU at 12 months, with a delta of 20%, and that the results herein presented are to be considered as a preliminary analysis only. Fourth, we did not implement a solicited reporting of AEs for mindfulness practice. Behavioral therapies are, however, considered to be less prone to side effects [19], and we neither recorded any interruption of treatment nor reported any relevant events during the sessions, such as panic attacks.

## 5. Conclusions

In conclusion, the findings of our preliminary analysis suggest the feasibility of implementing a mindfulness-based approach to the treatment of CM-MOH and provide initial support to the hypothesis of the superiority of such a treatment.

## Figures and Tables

**Table 1:** Baseline differences between the two groups.

	TaU (*n* = 88)	TaU + MIND (*n* = 89)	*p*
Age	47.6 ± 9.6	45.9 ± 11.1	0.285
Female sex	78 (87.6%)	79 (89.8%)	0.654
Duration of CM	15.9 ± 11.9	14.6 ± 10.1	0.431
Three-month headache frequency	63.5 ± 15.0	66.3 ± 15.6	0.225
Three-month total drug intake	119.3 ± 77.8	143.4 ± 117.3	0.108
Three-month NSAID intake	65.6 ± 69.0	96.6 ± 94.8	0.014
Three-month triptans intake	48.4 ± 55.1	44.6 ± 78.5	0.709

Notes. *t*-test for continuous variables, chi-squared test for categorical ones. For each variable where appropriate, means ± standard deviations were included. CM, chronic migraine; NSAIDs, non-steroidal anti-inflammatory drugs; TaU, treatment as usual.

**Table 2:** Repeated measures ANOVA between baseline and three months.

		Baseline	3-Month Follow-Up	Main Effect of Time		Time × Group Interaction		Time × Group Interaction with Age and CM Duration as Covariates	
				Wilks’ Lambda	*p*	Wilks’ Lambda	*p*	WILKS’ LAMBDA	*p*
Headache Frequency	TaU (*n* = 83)	63.1 ± 15.0	31.3 ± 20.3	0.29	<0.001	0.97	0.029	0.97	0.025
	TaU + MIND (*n* = 79)	66.3 ± 15.9	26.6 ± 21.8						
Total drug intake	TaU (*n* = 83)	118.1 ± 78.2	41.5 ± 40.5	0.54	<0.001	0.98	0.066	0.98	0.074
	TaU + MIND (*n* = 79)	145.7 ± 120.8	40.2 ± 35.8						
NSAIDs intake	TaU (*n* = 83)	63.4 ± 67.6	24.5 ± 32.6	0.65	<0.001	0.95	0.005	0.95	0.007
	TaU + MIND (*n* = 79)	96.8 ± 95.6	23.8 ± 25.9						
Triptans intake	TaU (*n* = 83)	49.0 ± 56.6	15.3 ± 19.2	0.81	<0.001	10.00	0.792	10.00	0.813
	TaU + MIND (*n* = 79)	47.0 ± 81.8	16.0 ± 19.7						

Notes. NSAIDs, non-steroidal anti-inflammatory drugs; TaU, treatment as usual.

## Data Availability

The data presented in this study are available on request from the corresponding author.
